# Chemical control for the morphogenesis of conducting polymer dendrites in water

**DOI:** 10.1039/d5lp00225g

**Published:** 2025-10-29

**Authors:** Antoine Baron, Corentin Scholaert, David Guérin, Yannick Coffinier, Fabien Alibart, Sébastien Pecqueur

**Affiliations:** a IEMN, UMR 8520 Univ. Lille, CNRS, Univ. Polytechnique Hauts-de-France 59000 Lille France sebastien.pecqueur@iemn.fr; b Laboratoire Nanotechnologies & Nanosystèmes (LN2) CNRS, Université de Sherbrooke J1X0A5 Sherbrooke Canada

## Abstract

Conducting polymer dendrite (CPD) morphogenesis is an electrochemical process that enables *in materio* evolving intelligence in wetware devices. During CPD morphogenesis, voltage transients drive the physical evolution of electrically conductive structures, thereby programming their filtering properties as nonlinear analog devices. Whether studied in an electrochemical experiment or in neuromorphic devices, the dependence of the electrical properties of the electrogenerated structures on the chemical composition of their growth environment is still unreported. In this study, we report the existing interconnection between the nature and concentration of the electrolytes, electroactive compounds and co-solvents and the electrical and electrochemical properties of CPDs in an aqueous electrolyte. CPDs exhibit various chemical sensitivities in water: their morphology is highly dependent on the nature of the chemical resources available in their environment. The selection of these resources therefore critically influences morphogenesis. In addition, the concentrations of the different electrochemical species have varying impacts on growth dynamics, modulating the balance between thermodynamic and kinetic control over polymer electrosynthesis. By correlating the dependencies of these evolving objects with the availability of the chemical resources in an aqueous environment, this study offers guidelines to tune the degree of evolution of electronic materials in water and highlights potential avenues for their application. Such evolving hardware is envisioned to exploit the chemical complexity of real-world environments as part of information processing technologies.

## Abstract 2

The relationship between the morphology of electrogenerated conducting polymer dendrites with the chemical composition of their environment and the adaptability of evolving polymers to water-based conditions for bio-inspired electronics are studied.

## Graphical abstract

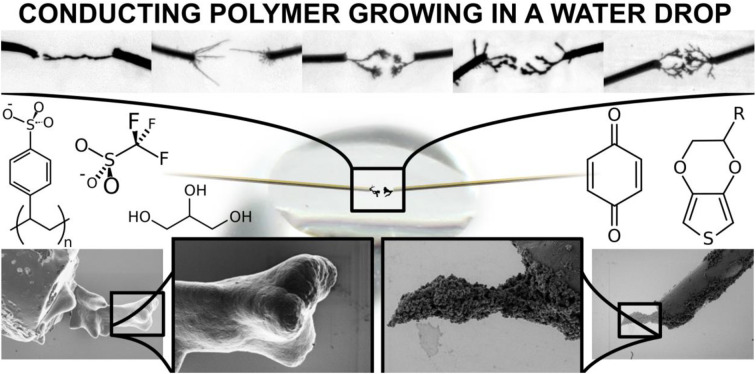

## 1 Introduction

The surge in activity driven by Internet-of-Things technology and large Artificial Intelligence models has led to a significant surge in energy and resource consumption.^1–5^ Moreover, electronic systems are routinely replaced due to damage or obsolescence,^6–8^ and their low recyclability contributes to pollution at a large scale.^9–11^ Although older electronics were modular, encouraging component reusability, current highly integrated systems incorporate nanoscale components that lack such flexibility.^12–14^ In contrast to software, which can adapt and upgrade itself, hardware remains fixed and must be designed *a priori* to meet the broadest needs, resulting in oversized circuits.^15^

Most of the aforementioned problems of conventional electronics stem from their fabrication, either due to resource-intensive manufacturing steps or the inherent rigidity of the materials.^16,17^ These challenges may be addressed by investigating novel materials for electronic components and interconnections. Conducting polymers, in particular, are promising for their low cost,^18,19^ high flexibility^20,21^ and ion-coupled electronic transport.^22,23^ Under a transient voltage, conducting polymer dendrites (CPDs) grow as fibrous structures and exhibit high versatility in aqueous environments, resembling the ramified structures found in nature.^24^ Their highly disordered structural features can be globally controlled by voltage. The mechanisms ruling their charge coupling with ions and their structural complexity provide many advantages for using CPD networks as a computing material.^25–28^ A train of low voltage spikes supplied between two electrodes can condition their growth to connect specific contacts,^29^ and when subjected to a dynamic voltage signal, their structural asymmetry is a relevant property for transient pattern classification.^28,30^ Finally, due to the electrochemical nature of their admittance, changing their morphology can modulate information transport by adapting their filtering properties.^31–33^

**Fig. 1:**
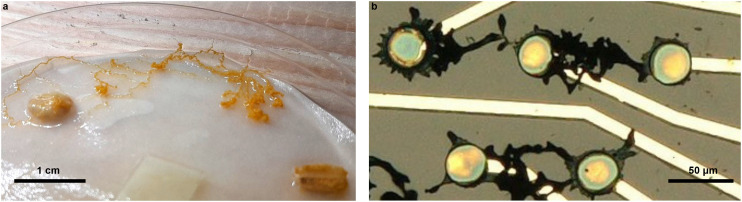
Structural analogy between slime mold and conducting polymer dendrites in water | (a) *Physarum polycephalum* growing in a Petri dish between oat flakes in water. (b) PEDOT:PSS growing on parylene C between voltage-supplied electrodes in water.

Their ability to change shape is a possible implementation of evolutionary electronics,^34^ which exploits *in operando* structural changes as a resource to process information.^35,36^ This enables autonomous, information-driven structural adaptation that supports vertical scaling, self-repair, energy management and dynamic allocation of computing and memory resources.^37–39^ This paradigm draws inspiration from nature, and the necessity of some biological systems to physically adapt their structure, from the brain’s topological plasticity to the networking behavior of sessile organisms.^40,41^ Some organisms, such as slime mold (Fig. 1a) have genuine abilities to solve classification tasks and have been proposed as low-resource computing materials in water.^42,43^

As a biocompatible medium, water is a promising platform for computing. Although computers have historically developed around hardware, liquids can also serve as substrates and take advantage of fluid dynamics to process information.^44^ Initially inspired by the brain as computing wetware,^45^ neuromorphic devices operating in water have been proposed as a physical implementation of reservoirs within the framework of reservoir computing. Because water is a universal substrate for biologically intelligent organisms, there is a necessity to carry out investigations on water-based machines to process and store information and communicate with other organisms in the same medium.

Up to now, CPDs have been mainly studied in acetonitrile, a volatile and often impractical organic solvent. However, the first tests reported in water in a comparative study with acetonitrile show that the morphology and behavior of CPDs can differ greatly.^46^ Overall, CPDs have been studied under only a limited number of very specific chemical conditions (Table 1). Their growth depends on the nature of the monomer, the redox-active counter agent, the electrolyte salt and the solvent, but quantitative correlations have not been performed. This study reports on the influence of the chemical composition of an aqueous electrolyte on the morphogenesis of CPDs, highlighting how changes in chemistry can guide structural features with particular applicative relevance.

**Table 1:** Growth conditions studied in the literature and in the present work

Monomer	Redox agent	Electrolyte	Solvent	Reference
500 mM pyrrole		125 mM	Aqueous	Dang *et al.* (2014)^47^
135 mM EDOT		20 mM	Aqueous	Dang *et al.* (2014)^47^
50 mM EDOT	5 mM BQ	1 mM Bu_4_NClO_4_	MeCN	Koizumi, Ohira *et al.*^48,49^
50 mM EDOT-CH_3_	5 mM BQ	1 mM Bu_4_NClO_4_	MeCN	Koizumi *et al.* (2016)^48^
50 mM EDOT-C_10_H_21_	5 mM BQ	1 mM Bu_4_NClO_4_	MeCN	Koizumi *et al.* (2016)^48^
50 mM EDOT	1 mM H_2_PtCl_6_		MeCN	Koizumi *et al.* (2018)^46^
50 mM EDOT	AgBF_4_		MeCN	Koizumi *et al.* (2018)^46^
10 mM EDOT		1 g L^−1^ NaPSS	H_2_O	Koizumi *et al.* (2018)^46^
10 mM EDOT		NaTs	H_2_O	Koizumi *et al.* (2018)^46^
150 mM EDOT	15 mM BQ	3 mM Bu_4_NClO_4_	MeCN	Watanabe *et al.* (2018)^50^
150 mM EDOT	15 mM BQ	3 mM Bu_4_NBF_4_	MeCN	Watanabe *et al.* (2018)^50^
150 mM EDOT	15 mM BQ	3 mM Bu_4_NPF_6_	MeCN	Watanabe *et al.* (2018)^50^
150 mM EDOT-CH_3_	15 mM BQ	3 mM Bu_4_NClO_4_	MeCN	Watanabe *et al.* (2018)^50^
150 mM EDOT-C_10_H_21_	15 mM BQ	3 mM Bu_4_NClO_4_	MeCN	Watanabe *et al.* (2018)^50^
150 mM EDOT-CH_2_Cl	15 mM BQ	3 mM Bu_4_NClO_4_	MeCN	Watanabe *et al.* (2018)^50^
150 mM bithiophene	15 mM BQ	3 mM Bu_4_NClO_4_	MeCN	Watanabe *et al.* (2018)^50^
50 mM EDOT	10 mM BQ	[Deme]BF_4_		Chen *et al.* (2023)^51^
50 mM EDOT	10 mM BQ	[Emim]BF_4_		Chen *et al.* (2023)^51^
50 mM EDOT	10 mM BQ	[Deme]NTf_2_		Chen *et al.* (2023)^51^
50 mM EDOT		1 mM Bu_4_NClO_4_	MeCN	Eickenscheidt *et al.* (2019)^52^
50 mM EDOT		1 mM Bu_4_NPF_6_	MeCN	Cucchi, Petrauskas *et al.*^26,53,54^
135 mM EDOT		20 mM “PSS”	H_2_O/MeCN	Akai-Kasaya, Hagiwara, Watanabe *et al.*^25,55–57^
10 mM EDOT	10 mM BQ	1 mM NaPSS	H_2_O	Janzakova, Scholaert, Baron *et al.*^24,27–33,58^

10 mM EDOT	10 mM BQ	μM–mM NaPSS	H_2_O	This work (Fig. 2)
10 mM EDOT	10 mM BQ	1 mM EmimOTf	H_2_O	This work (Fig. 3)
—–10 mM *x*EDOT + (1 − *x*)BQ—–		1 mM NaPSS	H_2_O	This work (Fig. 4)
10 mM EDOT	10 mM BQ	1 mM NaPSS	H_2_O/glycerol	This work (Fig. 5)
2.5–10 mM EDOT	10 mM BQ	1 mM NaPSS	H_2_O	This work (Fig. 6)
10 mM EDOT(g_4_)_*x*_	10 mM BQ	1 mM NaPSS	H_2_O	This work (Fig. 7)
10 mM EDOT(RSO_3_Na)_*x*_	10 mM BQ		H_2_O	This work (Fig. 8)

## 2 Experimental

### 2.1 Materials and methods

All purchased chemicals were used without further purification and were exposed to air, ambient light and room temperature. 3,4-Ethylenedioxythiophene (EDOT), glycerol, sodium polystyrene sulfonate (NaPSS, 70 kDa) and parabenzoquinone (BQ) were purchased from Sigma Aldrich. 1-Ethyl-3-methylimidazolium trifluoromethanesulfonate (emimOTf) was purchased from Solvionic. Sodium 4-(2,3-dihydrothieno[3,4-*b*][1,4]-dioxin-2-yl-methoxy)-1-butanesulfonate (EDOT(RSO_3_ Na) or EDOT-S)^59^ was purchased from BLD Pharm. 2-(2,5,8,11-Tetraoxadodecyl)-2,3-dihydro-thieno-[3,4-*b*][1,4]dioxine (EDOTg_4_) was synthesized as reported by Ghazal *et al.*^60^

### 2.2 CPD growth

Unless otherwise specified, CPDs were grown using a square wave voltage signal centered at 0 V, alternating between −4 V and +4 V (voltage amplitude *V*_p_ = 4 V and voltage offset *V*_off_ = 0 V) at a frequency of 80 Hz and a 50% duty cycle, generated by a 50 MS s^−1^ Dual-Channel Arbitrary Waveform Generator from Tabor Electronics. CPD growth was performed on 25 μm diameter gold wires purchased from Goodfellow, and placed on micromanipulators to control the inter-electrode distance, set to 240 μm.

### 2.3 Electrical characterization

Free-standing CPDs on gold wires were characterized *in situ* within the electrochemical environment in which they were grown: no electrode lithography nor CPD transfer was required. The electrical conductance of the dendrites was estimated by performing current–voltage (*I*–*V*) measurements with the help of an Agilent B1500A Semiconductor Analyzer coupled with a B2201A Switching Matrix. Impedance measurements were performed using a Solartron Analytical (Ametek) impedance analyzer, with the measurement taken between the shorted ends of the CPDs and a 25 μm diameter silver wire serving as a counter electrode. The transfer characteristic of the EDOT-S OECT was obtained by applying a voltage bias of 100 mV between the source and drain electrodes, with the source being grounded, and a sweeping voltage varying between −500 mV and +500 mV relative to the source was applied to the gate, with a hold time of one second and a delay of 100 ms. Voltage application was performed and current measurements were obtained using the SMU channels of a B1500A Semiconductor Device Analyzer.

### 2.4 Imaging

The optical microscope pictures presented throughout this paper were extracted from video footage of the growth captured using a VGA CCD color Camera from HITACHI Kokusai Electric Inc. Scanning Electron Microscopy (SEM) pictures were subsequently taken using the InLens detector of a Zeiss Ultra 55. The gold wires were carefully removed laterally from the electrolyte droplet to minimize capillary forces that could otherwise break the dendrites. The remaining structures were then transferred and fixed with tape onto a silicon substrate prior to imaging.

### 2.5 Potential modeling

Potential profiles across polarized CPDs are calculated according to the steady-state voltage drop yielded by the five resistive contributions in the presented compact model, taking into account the transfer resistances *R*^ox^_CT_ and *R*^red^_CT_ at both interfaces, the ion drift resistance 1/*G* of the electrolytic bulk between the electrodes, and the electrical resistance *R*(X^−^) and *R*(M^+^) of the electrogenerated dendrites. The different resistive contributions were either determined experimentally for each condition or assigned arbitrary values when deemed irrelevant. The electrical resistances *R*(X^−^) and *R*(M^+^) for each uncompleted CPD were considered to equal half of the measured electrical resistance determined *via* the (*I*–*V*) measurement of the completed CPD, considering their behavior to be ohmic (as characterized by Janzakova *et al.*^29^). The ion drift resistance 1/*G* of the electrolytic bulk between the electrodes was determined *via* impedance spectroscopy, using measurements performed between a third wire and the dendrite shorted at both ends (as performed by Janzakova *et al.*^58^).

## 3 Results

### 3.1 Media salinity and morphogenesis of conducting polymer dendrites

Regardless of the nature of the solvents or electrolytes employed, the growth of PEDOT-based CPDs is usually performed under mild saline conditions to promote electric-field activation in a medium behaving more as a dielectric than as an ion conductor (Table 1). Controlling the balance between the dielectric and conductive properties of water through salt concentration is thus expected to significantly influence CPD growth. In water, PSS is a polyanion extensively used in the literature because (1) it stabilizes the p-doping of PEDOT, (2) it promotes cation conduction in a solid PEDOT:PSS blend as an organic mixed ionic-electronic conductor (OMIEC)^61–65^ and (3) as an emulsifier, it stabilizes the hydrophobic PEDOT in water. Akai-Kasaya and coworkers reported that among different anions tested in a water/acetonitrile blend, PSS provided the highest degree of controllability and reproducibility, yielding thin and wire-like growths.^25^ However, the numerous roles PSS plays hinder the full understanding of its involvement during CPD growth.

**Fig. 2:**
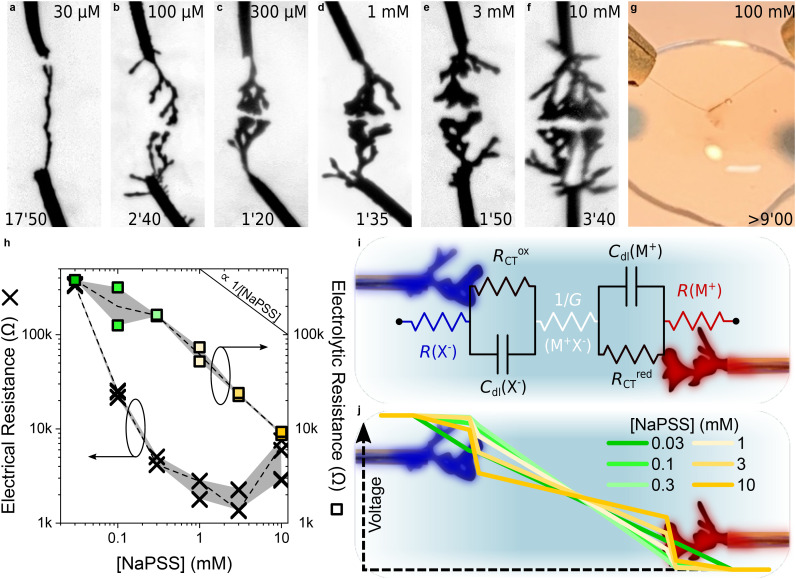
Influence of the concentration of NaPSS on CPD morphogenesis in water | (a–f) Optical pictures of CPD growths (4 *V*_p_, 0 *V*_off_, 80 Hz, 50%_dc_, 10 mM EDOT and 10 mM BQ) in aqueous media with different NaPSS concentrations (values displayed at the top of each picture). Each image was taken a few seconds before CPDs merged (applied-voltage duration displayed at the bottom of each picture). (g) Photograph of the electrochemical setup under the same experimental conditions as previously described, but with 100 mM NaPSS. Despite the absence of CPD between the wires, the presence of an electrogenerated material is visible. (h) Dependence of the electrical resistance (measured across each CPD displayed in Fig. 2a–f after they merged) and the electrolytic resistance (evaluated *via* impedancemetry at 10 kHz with a silver wire) on the concentration of NaPSS in the growth media. (i) 7-Element equivalent circuit highlighting the contribution of [NaPSS] on the ohmic resistance of the medium and the CPDs as both an electrolyte and a doping counter-ion carrier. (j) Simulated voltage drop according to the equivalent circuit presented in Fig. 3i, with *R*^ox^_CT_ = *R*^red^_CT_ = 10 kΩ, *R*(X^−^) = *R*(M^+^) defined as half the experimental value of the CPD electrical resistance once merged and 1/*G* as the experimental value of the electrolytic resistance measured with a silver wire. Blue indicates the point of highest potential in the circuit, whereas red indicates the point of lowest potential.

The morphological influence of the concentration of NaPSS can be observed in Fig. 2a–f, which present pictures of CPDs taken seconds prior completion at different salt concentrations, while all other parameters were fixed—the nature of the wires and of the electrolyte and the parameters of the voltage waveform. Fiber growth was found to occur for concentrations ranging from 30 μM to 10 mM. Higher and lower salt concentrations did not lead to dendritic growth experimentally, although it can be noted that, at 100 mM (Fig. 2g), a dark veil appeared around the electrodes and some material was deposited onto the gold wires, but no dendrites grew and bubbles were formed due to water electrolysis.

The morphology of the electrogenerated CPDs appeared to change with NaPSS concentration in the electrolyte. Lower concentrations resulted in more linear structures, whereas higher concentrations led to more voluminous and branched systems, with the appearance of the fibers gradually evolving with concentration. The duration of growth was also significantly affected by the concentration of NaPSS available in the medium, with a minimum of 100 seconds found to bridge a 240 μm gap with 1 mM NaPSS in water (Fig. 2e), while at 30 μM, the CPD took significantly longer to grow—it even required narrowing the gap during the nucleation phase to initiate growth on the gold surface.

CPDs can grow in ionic liquids,^51^ suggesting that the limitation observed at high NaPSS concentrations is not caused by the screening of the applied electric field due to an excessive ionic density. However, at these high concentrations, PEDOT might be too well dispersed in the medium, preventing CPDs from sticking on the gold electrodes as the anion behaves as a surfactant. Since NaPSS also operates as a doping counter-anion, excessive doping of PEDOT may result in highly charged particles, which in turn favors their suspension in the medium. These observations raise questions about the possibility of performing CPD growth in physiological environments, where salt concentrations reach such ranges, although this issue could be circumvented by leveraging the metabolic activity of living organisms to perform *in vivo* polymerization.^66–70^

The electrical properties of the electrogenerated objects and those of the medium were recorded at the end of completion. Fig. 2h presents the resistance of the dendrites calculated through *I*–*V* measurements and the resistance of the electrolyte estimated *via* impedancemetry. As expected, the conductivity of the solution increases quasi-linearly with salt concentration. More interestingly, the electrical resistance of the polymer fibers appears to decrease as the concentration of NaPSS increases up to 3 mM, before slightly increasing at 10 mM. Two effects might contribute. First, when salt concentration increases, the dendrites tend to become thicker, thus increasing their conductance. Second, since NaPSS acts as the dopant in PEDOT:PSS, increasing its concentration in the growth solution might increase the conductivity of the polymer. However, estimating the volume of these fractal objects to deduce conductivity values remains a challenge, making it difficult to separate the contributions of each effect. Yet, these results show that salt concentration is a parameter that influences the time it takes for the fibers to form, their morphology and the conductance of these objects.

The influence of salt concentration on CPD formation can be appreciated through the simulations of the voltage drops in the system presented in Fig. 2i and j. A low-complexity equivalent circuit was used to model the system, which consisted in a hole transport resistance *R* for each end of the CPD, a double layer capacitance *C*_dl_ in parallel with a charge-transfer resistance (*R*^ox^_CT_ and *R*^red^_CT_) that models the redox reactions at the two polymer/electrolyte interfaces, and the electrolytic resistance 1/*G* governed by ion transport in the medium (Fig. 2i). As a first-level approximation, it was assumed that the resistance of each uncompleted CPD was independent of the applied voltage and corresponded to half of the measured electrical resistance displayed in Fig. 2h. It was also considered that the electrolytic resistance 1/*G*, which was limited by the ion drift between two CPDs, was also representative of the impedance between the electrodes and the silver wire used for the impedance measurement. Additionally, both *R*_CT_ values were fixed at 10 kΩ and both *C*_dl_ values were considered to correspond to ideal Debye capacitors. As presented in Fig. 2j, the results show that the concentration of NaPSS influences the voltage drops in the system through the modulation of both the electrolytic resistance and the electrical resistance of the dendrite. For low salt concentrations, the voltage drop across the fibers cannot be neglected because of the low conductance of CPDs. As a consequence, the potential is not uniformly distributed along the fibers. The voltage drop at the electrode/electrolyte interfaces increases with the concentration of NaPSS, therefore driving electropolymerization at the tip of the electrodes. Despite the morphological influence of salt concentration, these results confirm that two antagonistic effects—both governed by the presence of salt in the medium—interplay to control growth kinetics through their influence on the voltage drop: insufficient salt leads to poorly conductive CPDs, while excessively high salt concentrations result in an overly conductive electrolyte.

The impact of the ions on CPD morphology has been explored using different ionic liquids (ILs) where clear morphology differences were observed depending on ion diffusion coefficients,^51^ but the high viscosity of these media makes it unclear whether the differences come from ion chemistry or the medium's viscosity.

Based on Table 1, CPDs mainly grow under conditions that favor a transport-limited current (high viscosity or low ionic concentration). This means that counter ions other than PSS may also work if sufficiently diluted and with a mobility compatible with the medium's viscosity. In water, the low viscosity results in high ion mobility, so that diffusion-limited growth is reached at low anion concentration and high potential. In contrast, the case of Fig. 2g can be seen as reaction-limited due to a high ionic concentration.

As EmimOTf is an IL miscible in water and previously used to form the PEDOT:OTf polymer,^71^ it raises the question whether triflate ions (OTf^−^) can also enable CPD growth in water, and if the resulting CPDs are different depending on the nature of the counter ion. PEDOT:PSS and PEDOT:OTf CPDs were compared to address these questions. Both electrolytes were introduced at 1 mM, previously established as the optimal concentration for fast growth, with 10 mM EDOT and BQ in water. The applied growth signal was identical in both cases, making the doping anion the only difference between the two experiments. While using NaPSS leads to thick branches, EmimOTf produces thinner branches that grow very quickly, taking only around 15 seconds to complete compared to more than a minute for PEDOT:PSS.

It is hypothesized that the observed differences mainly arise from differences in ionic mobility. In the case of the NaPSS salt, PSS is a polyanion with high molecular weight (70 kDa used in the present study), while triflate ions only carry a single charge. Since the growth medium is water, the anion drift is hindered by friction, which increases with molecular size. PSS has one negative charge per monomer, but counter-ion condensation can lower its effective charge, yielding a large molecule with reduced charge density.^72^ Therefore, the faster growth dynamics of triflate ions could be explained by their greater mobility. PEDOT:OTf CPD growth follows the paths of least resistance dictated by the field between the two wires, leaving less time for diffusive and hydrodynamic effects to alter the ions’ trajectory. Moreover, because of their high mobility, OTf^−^ ions travel farther than PSS ions in the same time, explaining why branches can grow from the sides of the wires.

**Fig. 3:**
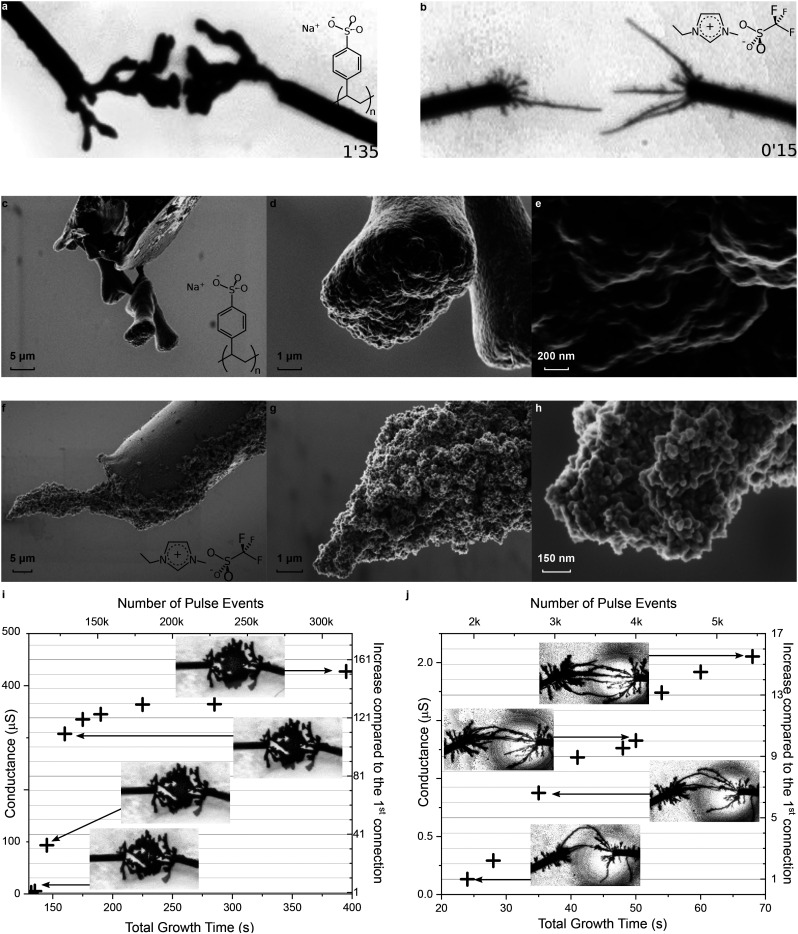
Influence of the nature of the salt on CPD morphology in water | (a) Optical picture of a CPD grown at 4 *V*_p_, 0 *V*_off_, 80 Hz, and 50%_dc_ in water with 10 mM EDOT and 10 mM BQ at 1 mM NaPSS after 95 seconds of applied voltage. (b) Optical picture of a CPD grown under exactly the same conditions as in (a), but using EmimOTf instead of NaPSS as an electrolyte and after only 15 seconds of applied voltage. (c–e) Scanning electron microscopy (SEM) images of a CPD grown under the same conditions as displayed in (a) with NaPSS as an electrolyte. (f–h) SEM images of a CPD grown under the same conditions as displayed in (b) with EmimOTf as an electrolyte. (i and j) Filamentary switching: (i) shows the evolution of conductance for PEDOT:PSS CPDs over time, starting after the initial connection. Each sample approximately corresponds to the connection of a new branch. (j) Shows the same experiment for PEDOT:OTf CPDs. Insets are black and white optical microscopy pictures of the corresponding CPDs at different times.

The resulting CPDs have been compared in a dried state *via* scanning electron microscopy. Fig. 3c–e show the same PEDOT:PSS CPD observed at different magnifications. First, the images confirm that deposition on the sides of the wire is limited, with most of the growth occurring at the tip. The sides of the dendritic structure appear smooth, whereas the tip displays multiple nucleation sites and a higher roughness overall. This roughness is not the result of the CPD breaking during sample handling. The tip's frontal surface is not uniform: a central depression is flanked by protruding regions, suggesting that after a critical growth time, the surface splits and one branch becomes favored depending on the path of least resistance. The surface of the inactive branch eventually smoothens, possibly due to swelling from water absorption or the surfactant effect of PSS ions.

On the other hand, PEDOT:OTf CPDs (Fig. 3f–h), made from the same monomer in the same solvent, appear more granular. The grains are smaller than 50 nm and not bound together by a binder such as PSS. Although this high roughness provides multiple potential nucleation sites, the main branches rarely split, in contrast to PEDOT:PSS.

This difference in growth modes can be employed in various applications, such as filamentary switching, which involves modulating the conductance of a connection through the formation and rupture of conductive filaments.^73,74^ After the initial completion of the dendritic connection, the same growth signal is reapplied and *I*–*V* measurements are performed at different times to evaluate the possible irreversible modulation of conductance after connection. In the case of PEDOT:PSS dendrites, sudden conductance jumps as high as 200 μS can occur, resulting from the growth of inner branches at the junction. The large PEDOT:PSS branches connecting together results in a higher electronic current increase because of the sudden gain in active surface area, and gradually, the connection is reinforced by branches merging together around the junction, eventually forming a single branch if given enough time.

On the other hand, the PEDOT:OTf CPDs show a more linear increase in conductance due to the continuous connection of new branches between the two electrodes. Side branches, considered “dead” before the connection, are developing again and contribute to strengthening the connection either ionically or electronically when connecting to the nearest branches, possibly to one belonging to the same side of the system. The conductance of PEDOT:PSS, highly dependent on the active surface, can reach two orders of magnitude higher than that of PEDOT:OTf.

By changing the nature of only the counter ion, it is possible not only to get different conductivities for the material but also to modify the modulation of conductance and its dynamics to better suit specific applications.

### 3.2 Electrochemical control of growth velocity *via* charge-transfer balance

The electropolymerization of EDOT into PEDOT is an oxidation reaction that releases protons in the medium, thus requiring a counterbalancing reduction reaction to respect electroneutrality. This aspect is often neglected, and the reduction is typically left to the solvent.^54,57^ However, controlling growth calls for a complete understanding of both the anodic and the cathodic reaction. Even when water was used as the solvent, no gas production was evidenced, suggesting that Nernst thermodynamic potentials are not reliable predictors of faradaic involvement of solvent molecules during CPD morphogenesis—including when the voltage amplitude exceeds the electrochemical window of the solvent. In this regard, Inagi and coworkers introduced *p*-benzoquinone (BQ) in acetonitrile to behave as a sacrificial reagent for the redox reaction and avoid pH changes due to the release of protons during electropolymerization.^48^ Here, we propose investigating the impact of the BQ : EDOT ratio on the growth and morphology of CPDs in water. To do so, the total amount of electroactive material in the medium was kept constant throughout this series of experiments ([BQ] + [EDOT] = 10 mM), so that the thermodynamics of the reaction would remain as similar as possible while the influence of BQ was under study.

**Fig. 4:**
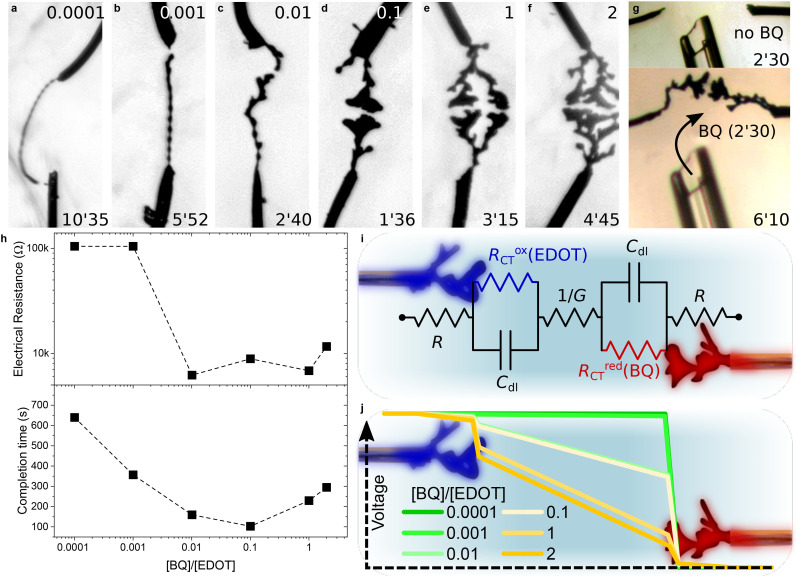
Influence of BQ as an electroactive counter agent for CPD morphogenesis in water | (a–f) Optical pictures of CPD growth (4 *V*_p_, 0 *V*_off_, 80 Hz, 50%_dc_, 10 mM [EDOT + BQ] and 1 mM NaPSS) in an aqueous medium with different [BQ]/[EDOT] molar ratios (values displayed at the top of each picture). Each image was taken a few seconds before the CPDs merged (applied-voltage duration displayed at the bottom of each picture). (g) Introduction of a BQ aqueous solution, triggering CPD growth in an aqueous solution containing 10 mM EDOT and 1 mM NaPSS. (h) Dependence of the electrical resistance (measured across each CPD displayed in (a–f) after they merged) and their growth duration on the [BQ]/[EDOT] molar ratio contained in the growth media. (i) 7-Element equivalent circuit highlighting the contributions of [EDOT] and [BQ] to the charge-transfer balance between oxidation and reduction. (j) Simulated voltage drop according to the equivalent circuit presented in (i), with *R* defined as half the experimental value of the CPD electrical resistance once merged, 1/*G* = 60 kΩ, *R*^ox^_CT_ = 100 kΩ mM_EDOT_^−1^ and *R*^red^_CT_ = 100 kΩ mM_BQ_^−1^. Blue indicates the point of highest potential in the circuit, whereas red indicates the point of lowest potential.

The influence of the BQ : EDOT ratio on CPD growth—particularly on morphology and completion time—is shown in Fig. 4a–f. Even small amounts of BQ (1 : 10 000 and 1 : 1000 BQ : EDOT) enabled dendritic formation, in the form of thin and linear fibers that took longer to form. Interestingly, these dendrites grew mainly out of one of the two electrodes of the system in a rosary-like fashion, rather than the simultaneous growth from both electrodes that is usually observed. As the ratio increases up to 1 : 10, the completion time decreases and the objects progressively become more voluminous. For higher BQ contents, the dendrites keep getting more ramified. The increase in completion time observed for the 1 : 1 and 2 : 1 BQ : EDOT ratios might be due to the associated decrease in the monomer concentration ([BQ] + [EDOT] = 10 mM) rather than the effect of ratio modulation itself, since the low solubility of EDOT in water prevented us from testing higher ratios under these experimental conditions.

Understanding the influence of the electrochemical environment is a valuable asset in controlling growth, as illustrated by Fig. 4g, where the usual experimental setup was used, except that BQ was removed from the system. In the absence of a sacrificial reagent, no growth was observed for more than two minutes, although a signal was applied between the two electrodes. BQ was then gently introduced into the system with the help of a capillary, triggering growth. This experiment suggests that CPD growth could be controlled by fine-tuning the electrochemical medium, locally introducing small amounts of certain chemical species. This feature could be critical for growth in specific environments such as biological ones, as many molecules (including BQ) are not biocompatible.

The electrical measurements displayed in Fig. 4h once again highlight the dependence of the electrical resistance of the polymer fibers on their morphology: linear dendrites ([BQ]/[EDOT] ≤ 0.001) present a higher electrical resistance than more voluminous and ramified fibers ([BQ]/[EDOT] ≥ 0.001). A strong correlation can also be observed between the completion time of the CPDs and the BQ : EDOT ratio with a marked decrease as the ratio increases, although the increasing completion time for the last two points of the graph might be related to the low concentration of monomer in the solution.

The equivalent circuit presented in Fig. 4i helps in understanding how the BQ : EDOT ratio influences the electropolymerization process. During growth, a redox reaction happens at the electrode/electrolyte interfaces. In the equivalent circuit, this faradaic reaction is represented as a charge-transfer resistance. The influence of BQ and EDOT concentrations was assumed to be linear with respect to the charge-transfer resistance, such that *R*^ox^_CT_ = 100 kΩ mM_EDOT_^−1^ and *R*^red^_CT_ = 100 kΩ mM_BQ_^−1^. Fig. 4j shows that for very low concentrations of BQ, the charge-transfer resistance at the cathode is extremely high, therefore causing most of the voltage to drop at the cathode/electrolyte interface. As [BQ]/[EDOT] increases, so does the value of *R*^red^_CT_/*R*^ox^_CT_, causing the voltage drops in the system to distribute more evenly, facilitating electropolymerization. Therefore, the impact of BQ on CPD growth is similar to that of NaPSS (see Fig. 2), since they both influence voltage drops at the interfaces, but through different mechanisms. However, growth was still observed at low concentrations of BQ. A possible explanation is the presence of trace contaminants that originate from the substrate, the electrolyte preparation, or the ambient environment. Experimentally, limiting the availability of BQ is a serious constraint for CPD growth, as the fibers grow slower and less bulky.

These results confirm that introducing BQ as an oxidizing agent in trace amounts may be sufficient to trigger CPD growth. Even in water, the lack of control in the concentration of BQ as an oxidizing counter agent has an important influence over growth kinetics and the resulting CPD morphologies.

### 3.3 Electrokinetic control of morphogenesis *via* viscosity variation

**Fig. 5:**
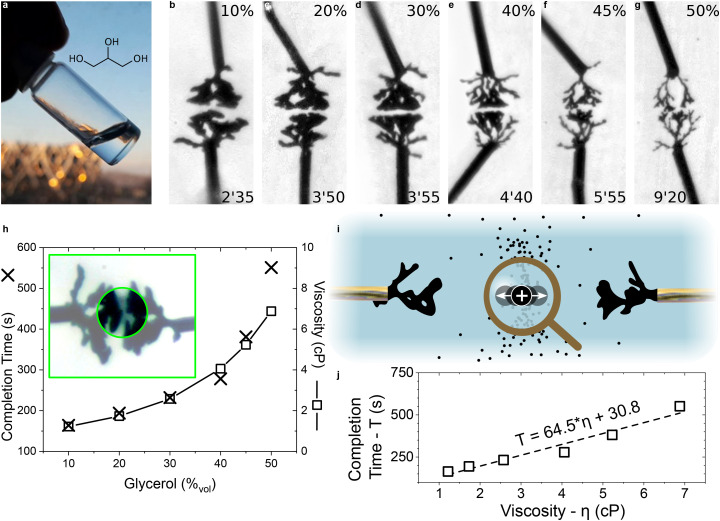
Influence of glycerol as a higher-viscosity co-solvent on CPD morphogenesis | (a) Image of pure glycerol as a small-molecule non-acidic hydroxylated solvent.^84,85^ (b–g) Optical pictures of CPD growth (5 *V*_p_, 0 *V*_off_, 40 Hz, 50%_dc_, 10 mM EDOT, 10 mM BQ and 1 mM NaPSS) in aqueous media with different ratios of glycerol (values displayed at the top of each picture). Each image was taken a few seconds before the CPDs merged (applied-voltage duration displayed at the bottom of each picture). (h) Dependence of the completion time on the volume content of glycerol (the trend is compared to viscosity values of glycerol/water mixtures calculated from Cheng *et al.*).^82^ Inset: contrasted image of the 10% growth where flowing particles were evidenced. (i) Schematic of a growth mechanism driven by the distribution of positively-charged conducting-polymer particles. (j) Correlation between completion time and the viscosity values displayed in (h).

The influence of the counter ion on the morphology and growth dynamics of CPDs was discussed earlier. These changes affect the electrical properties of the connection, which motivates a closer investigation of the underlying physics. The mobility of the species, identified as a key parameter, can be controlled through the medium's viscosity. Since signs of electrokinetic effects (electrophoresis or electroconvection) appear near the voltage where CPD growth begins (∼eq 3.5 V, 80 Hz in water),^24^ changing the solvent's viscosity is expected to influence the growth mechanism. A high viscosity also hinders the formation of electrohydrodynamic instabilities^75,76^ that have been suspected to be involved in the formation of metallic dendritic deposits.^77^ Moreover, viscous media are often easier to integrate into complex systems due to their reduced volatility and more controlled flow.^78,79^ In water, viscosity can be modulated by adding a fraction of glycerol (Fig. 5a), a non-toxic, non-acidic, hydroxylated small molecule with a viscosity more than a thousand times higher than water.^80^ It is also freely miscible with water,^81^ and mixtures of water/glycerol have been well-studied in the literature, allowing viscosity values for different ratios to be estimated using existing models.^82^ Finally, its addition should not significantly alter the solvent's chemistry.

CPD growth at different ratios of glycerol/water was conducted using the same concentration of monomer and ions as in the previous experiments (1 mM NaPSS, 10 mM EDOT). Pictures of the resulting CPDs are presented Fig. 5b–g. A progression in the morphology is visible: for low ratios, branches are thicker. Close to completion, branches grow at the same pace, very close to each other. In this situation, oligomer particles can be seen transiting between the two tips. A clear shift in morphology starts to appear around 40%, where the thickness is notably reduced. The time taken for the CPDs to connect to each other was recorded and plotted in Fig. 5h against the ratio of glycerol in solution. It shows a non-linear increase in completion time that decently matches the increase of viscosity obtained for different mixtures of glycerol/water using the model of Cheng *et al.*,^82^ directly linking dendritic growth to viscosity.

The viscosity change between 40% and 50%, which corresponds to the largest morphological changes in CPDs, is also substantial compared to viscosities below 30%. Kumar *et al.*^83^ showed that at low electrolyte concentrations, charged particles under an AC field between two rectangular electrodes gradually concentrate at the center of the gap. These charged particles can be oligomers whose electropolymerization reaction was interrupted by the periodic change of sign of the AC electric field, or parts of the already grown oxidized CPDs detached from the CPDs under the existing electric and hydrodynamic stresses. As shown in the inset of Fig. 5h, for thick CPDs close to completion, those particles are confined in the gap and appear as a black cloud oscillating between the electrodes. Their back-and-forth motion is depicted in Fig. 5i and is thought to be a form of AC-field-driven electrophoresis^48^ of oligomers either dispersed or dissolved in solution.

For particles confined in the gap, an increased viscosity means that statistically fewer particles will reach the tips of the CPD for the same period of time, leading to thinner branches. This thin morphology is similar to the one observed for high growth signal frequencies, except that adding glycerol as a co-solvent leads to slower growth. Fig. 5j shows a clear correlation between the viscosity of the solution and the completion time of the CPDs, suggesting that the completion time could be predicted to some degree. However, it is important to stress that the completion time exhibits variability, as reported in water.^29^

In conclusion, viscosity limits the range of achievable morphologies to thin CPDs, but does not completely prevent growth. These results stress the particular challenge of enabling CPD morphogenesis in solid or colloidal electrolytes such as hydrogels.

### 3.4 Influence of the concentration of electroactive monomers on morphogenesis

**Fig. 6:**
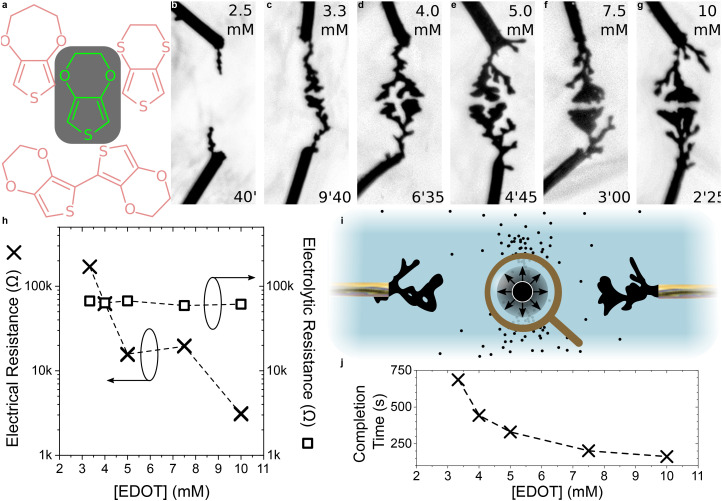
Influence of the concentration of EDOT on CPD morphogenesis | (a) EDOT as a poorly but yet significantly more water soluble monomer than other structurally similar derivatives, such as 3,4-propylenedioxythiophene, 3,4-ethylenedithiathiophene and the dimer of EDOT, which are insoluble in water. (b–g) Optical pictures of CPD growth (4 *V*_p_, 0 *V*_off_, 80 Hz, 50%_dc_, 10 mM BQ and 1 mM NaPSS in water) with different molar concentrations of EDOT (values displayed at the top of each picture). Each image was taken a few seconds before the CPDs merged (applied-voltage duration displayed at the bottom of each picture). (h) Dependence of the electrical resistance (measured across each CPD displayed in (b–g) after they merged) and the electrolytic resistance (evaluated *via* impedancemetry at 10 kHz with a silver wire) on the concentration of EDOT in the growth media. (i) Schematic of a growth mechanism limited by the concentration of EDOT where the kinetics of the reaction are influenced by the particle density and size distribution in the medium. (j) Dependence of the completion time of each CPD displayed in (b–g) on the concentration of EDOT in the growth media.

In electrochemistry, water is often disregarded as a solvent given its short electrochemical window. Its proticity induces pH-dependence in redox equilibria, necessitating the use of a buffer to maintain stability during the anodic electropolymerization of proton-releasing thiophene-based monomers. Additionally, many of these organic molecules are poorly soluble in water, thus requiring chemical engineering strategies to promote their hydrophilicity.^59,60,86–89^ In this regard, EDOT is an exceptional candidate: in contrast to its dimer, its three-carbon dioxyalkylated analog, or its ethylenedithia equivalent (Fig. 6a), EDOT can be solubilized in pure water up to about 15 mM at 25 °C.^90^ Watanabe *et al.* demonstrated that a threefold more concentrated medium affects the morphology of electrogenerated fibers.^50^ However, the specific influence of monomer concentration on growth has never been characterized, despite the direct impact of EDOT availability on the formation of PEDOT-based CPDs. Given the low solubility of EDOT in water, even in the presence of a surfactant, this parameter is therefore of particular importance. Investigating CPD morphogenesis in water with respect to monomer concentration is necessary to assess the feasibility of its implementation on water-based substrates.

Using the experimental setup described above, six dendrites were grown at different concentrations of EDOT (Fig. 6). Fig. 6b–g show that, as the quantity of monomer increases, the electrogenerated objects tend to become more ramified, although this effect is less pronounced than in the case of NaPSS (Fig. 2) or BQ variations (Fig. 4). Due to the low solubility of EDOT in water, only a narrow concentration range was attainable, which could partly explain the lack of noticeable morphological differences. The impact of monomer concentration on growth is best observed when looking at the completion time of the fibers, which decreases as the concentration of EDOT increases. When lower than 2.5 mM, the fibers could not merge after 40 min of growth, whereas the dendrites formed within just 145 s in the presence of 10 mM of EDOT. Meanwhile, Fig. 6h shows that the electrical resistance of the fibers tended to decrease as monomer concentration increased. Once again, this observation is best explained by morphological differences rather than by an increased conductivity: since the electrolytic resistance remains constant throughout the set of experiments (the concentration of NaPSS is set to 1 mM), the doping level is expected to remain equivalent in all cases, and so should conductivity.

The PEDOT growth rate would be expected to be directly proportional to the available quantity of monomer in the solution. Yet, the completion time increases exponentially as [EDOT] decreases (Fig. 6j). As the amount of BQ in the system is set to 10 mM, this parameter should not limit the reaction, as investigated in Fig. 4. Interestingly, and assuming the concentrations of electrolyte and BQ remain constant, lowering the concentration of EDOT should further increase the voltage drop at the anode interface, therefore promoting EDOT oxidation through an increased voltage bias in a two-electrode configuration. The concentration of monomer could impact the density and size distribution of the particles in the environment, thus electrokinetically limiting the reaction (Fig. 6i). The relationship between EDOT concentration and the colloidal properties of the medium as a suspension of electrogenerated PEDOT particles is not obvious and would require simulations. However, it appears to explain the superlinearity of CPD growth with EDOT concentration by the proximity of PEDOT particles, which influences their probability of coalescing, indirectly impacting CPD growth on the gold wires.

**Fig. 7:**
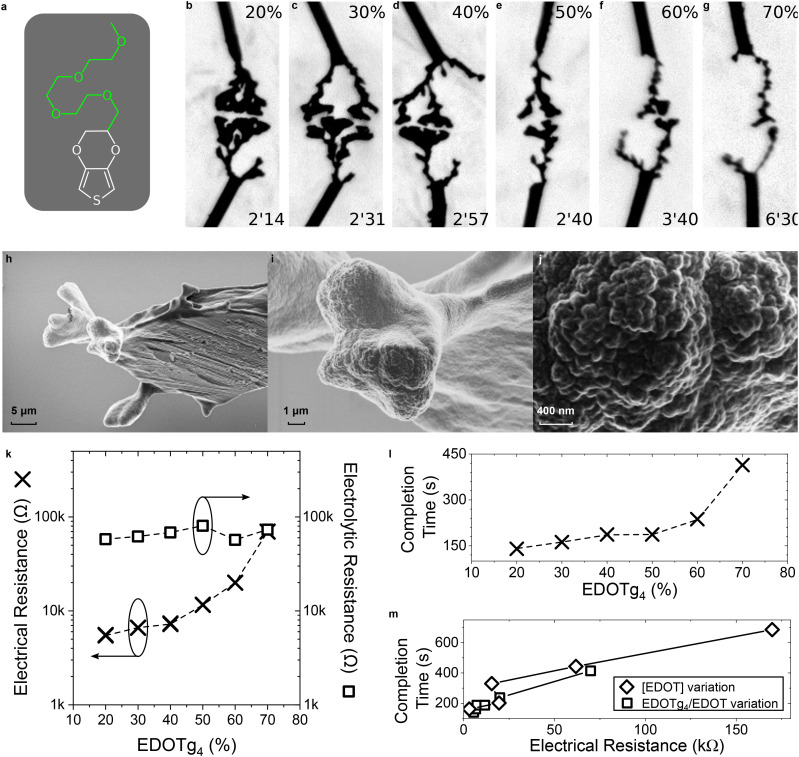
Influence of increased monomer hydrophilicity with EDOTg_4_ on CPD morphogenesis in water | (a) Chemical structure of EDOTg_4_, as a structural analogue of EDOT with a hydrophilic side chain, synthesized as already reported by Ghazal *et al.*^60^ (b–g) Optical pictures of CPD growth (4 *V*_p_, 0 *V*_off_, 80 Hz, 50%_dc_, 10 mM of total monomer content, 10 mM BQ and 1 mM NaPSS in water) with different molar ratios of EDOTg_4_ copolymerized with EDOT (values displayed at the top of each picture). Each image was taken a few seconds before the CPDs merged (applied-voltage duration displayed at the bottom of each picture). (h–j), SEM images of a CPD grown under the same conditions as displayed in (g). (k) Dependence of the electrical resistance (measured across each CPD displayed in (b–g) after they merged) and the electrolytic resistance (evaluated *via* impedancemetry at 10 kHz with a silver wire) on the molar ratio of EDOTg_4_ copolymerized with EDOT. (l) Dependence of the completion time on the molar ratio of EDOTg_4_ copolymerized with EDOT. (m) Comparison between the influence of EDOT dilution caused by EDOTg_4_ substitution, at 10 mM total monomer content, on growth latency and electrical resistance, and the results obtained in Fig. 6 for EDOT variation only.

The challenge imposed by the low solubility of thiophene-based monomers in water may be addressed by chemically engineering the molecules. As shown by Koizumi *et al.*,^48^ morphology can be controlled by modifying the chemistry of an EDOT monomer substituted on the ethylenedioxy bridge, thereby changing its affinity with the environment without altering its electroactivity. Under the same conditions, less dendritic and longer-branched growths were promoted with a –C_10_H_21_ alkyl chain substitution than with a –CH_3_ substitution. Yet, this effect might not be observed with every substitute of EDOT. A derivative of EDOT with a glycolated side chain, EDOTg_4_,^60^ was introduced to be co-electropolymerized alongside EDOT (Fig. 7a). The total amount of monomer in the solution was kept constant ([EDOT] + [EDOTg_4_] = 10 mM), while the proportion of EDOTg_4_ in the solution was increased. Fig. 7b–g show optical pictures of polymer fibers grown in the presence of both EDOT and EDOTg_4_, from 20% EDOTg_4_ to 70% EDOTg_4_. As can be observed, the electrogenerated fibers tended to become more linear as the proportion of EDOTg_4_ in the solution increased, while the completion time of the dendrites also increased. In contrast to the experiments performed by Koizumi, Watanabe *et al.* on the effect of EDOT substitution,^48,50^ no growth was observed when EDOTg_4_ was used as the only monomer. This correlates with Ghazal *et al.*'s results showing that DC electro-co-polymerization of EDOT with EDOTg_4_ above 70% EDOTg_4_ produces a polymer that is too hydrophilic to remain on the electrodes.^60^ As a result, it is difficult to attribute the change in morphology to either the presence of EDOTg_4_ or the dilution of EDOT, as the latter has been shown to impact morphology. The fibers did not present any visual clue of the presence of EDOTg_4_ within the polymerized structure. The SEM images displayed in Fig. 7h–j also confirm that the morphology of these fibers does not differ much from that of dendrites grown only in the presence of EDOT (see Fig. 3c–e). In addition, the electrical resistance of these structures tended to increase with the amount of EDOTg_4_ in the medium, whereas the electrolytic resistance remained constant, thus discarding the possibility of a decrease in conductivity (Fig. 7k). The completion time of these dendrites also importantly increased with the [EDOTg_4_]/[EDOT] ratio (Fig. 7l). Therefore, the presence of EDOTg_4_ within the electropolymerized dendrites cannot be confirmed. Moreover, the results discussed above are highly similar to those obtained when only decreasing the concentration of EDOT, notably the electrical resistance and completion time of the fibers, as can be observed in Fig. 7m. EDOTg_4_ could act only as a spectator, showing that modifying the chemistry of the monomer might not be sufficient to influence dendritic development.

In addition to the solvent, the CPD growth conducted so far always involved three ingredients: a monomer, an electrolyte and a redox agent. Using complex media with multiple components can create different sources of variability that can all impact growth. To address these challenges, efforts have been made to simplify the composition of the medium with components that can assume multiple roles at the same time.

**Fig. 8:**
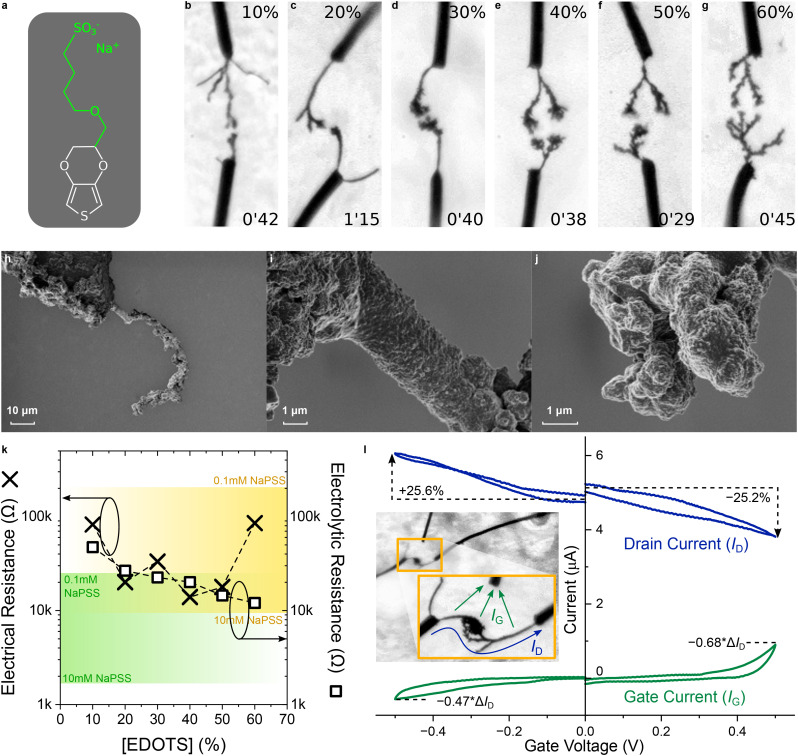
Influence of EDOT-S as an electroactive conjugated polyelectrolyte precursor on CPD morphogenesis in water | (a) Chemical structure of EDOT-S. (b–g) Optical pictures of CPD growth (4 *V*_p_, 0 *V*_off_, 80 Hz, 50%_dc_, 10 mM of total monomer content and 10 mM BQ in water) with different molar ratios of EDOT-S copolymerized with EDOT (values displayed at the top of each picture). Each image was taken a few seconds before the CPDs merged (applied-voltage duration displayed at the bottom of each picture). (h–j) SEM images of a CPD grown under the same conditions as displayed in (f). (k) Dependence of the electrical resistance (measured across each CPD displayed in (b–g) after they merged) and the electrolytic resistance (evaluated *via* electrochemical impedance spectroscopy at 10 kHz with a silver wire) on the molar ratio of EDOT-S copolymerized with EDOT. (l) Transfer characteristic of the CPDs displayed in (b–g) in a merged state, using a silver wire as a gate electrode (as displayed in the inset).

First, the electrolyte can act as a solvent itself, like in the case of ionic liquids. When the salt is also an oxidizing agent, the electrolyte can even substitute BQ as a redox agent, such as H_2_PtCl_6_.^46^ Here, the use of a conjugated polyelectrolyte precursor acting as both a monomer and an electrolyte is investigated. Sodium 4-(2,3-dihydrothieno[3,4-*b*][1,4]-dioxin-2-yl-methoxy)-1-butanesulfonate (EDOT(RSO_3_ Na)) or EDOT-S is an electroactive EDOT monomer derivative, carrying a sulfonate-terminated alkoxy chain on the covalently attached ethylene dioxy bridge (Fig. 8a).^59,91,92^ This leads to a greater solubility of the monomer in water compared to regular EDOT, with a sulfonate group carrying a negative charge to support the polymer p-doping. The sulfonate-terminated charge on EDOT-S may have a greater impact than that obtained with a neutral glycol chain on EDOTg_4_.

Similarly, EDOT-S was electro-co-polymerized with EDOT at various EDOT-S ratios, maintaining a total monomer concentration of 10 mM and including benzoquinone at 10 mM. No extra salts were involved. Thus, the electrolyte was composed of EDOT-S at different concentrations, supported by sodium cations. CPD morphology appeared to depend on the ratio of EDOT-S involved during electro-co-polymerization (Fig. 8b–g). At 10% (10 mM total monomer, including 1 mM EDOT-S in Fig. 8b), the branches were thinner and more linear compared to 10 mM EDOT growing in 1 mM NaPSS (Fig. 2f). This demonstrates that sulfonate anchoring on monomers significantly alters morphogenesis. At any EDOT-S : EDOT ratio, CPDs grew significantly faster than with EDOT only. The fast, linear growth and thin branch thickness is more similar to the growth behaviour of PEDOT:OTf CPDs (Fig. 3b). However, a notable difference is the absence of branches on the sides of the wires. In some cases, particularly for 30% and 40% EDOT-S, a multitude of short and thin branches formed at the tip of the CPDs. At higher ratios, the entire CPD appeared thicker and rougher. Beyond 60% EDOT-S, bubbles started to appear and no growth was observed. Indeed, attempting to electrochemically form an EDOT-S homopolymer is notoriously difficult in water, as was first observed by Stéphan *et al.*,^91^ where its electropolymerization only resulted in soluble oligomers diffusing away from the electrodes.

SEM images of EDOT-S CPDs grown for a ratio of 50% of each monomer are shown in Fig. 8h–j. The coating on the wire presents a high roughness, also visible on the curved stem of the CPD. During growth, the CPD transitioned from a smooth surface to a more curved and rough morphology. The InLens signal gives differences of brightness between parts of the CPD that could potentially relate to local differences in conductivity.

The electrical and electrolytic resistances were measured at the end of CPD growth, for the different proportions of EDOT and EDOT-S (Fig. 8k). As expected, the electrolytic conductance is controlled by the concentration of EDOT-S. In contrast, the electrical resistance does not vary monotonically with the ratio of EDOT-S : EDOT. There appears to be an optimum: low EDOT-S concentrations reduce CPD conductance, while high concentrations may increase the fragility of the structure in water due to the presence of charged particles in a polar solvent. The lowest resistance, achieved for 40% EDOT-S, is still an order of magnitude higher than that of EDOT-based CPDs grown with 1 mM NaPSS under the same conditions. As an OMIEC,^93–95^ a balance between electric and ionic conduction may control the electrochemical behavior of CPDs incorporating EDOT-S. PEDOT CPDs act as organic electrochemical transistors (OECT) in aqueous electrolytes.^30,54,58^ A similar behavior has also been evidenced in the case of CPDs copolymerized using EDOT and EDOT-S without involving any other salts (Fig. 8l). As a conjugated polyelectrolyte, PEDOT-S has an inherent OECT effect.^96–100^

This effect was tested using a three-electrode setup consisting of two gold electrodes short-circuited by an EDOT-S/EDOT CPD, and a silver wire serving as a gate electrode, positioned in close proximity to the junction between the two CPDs. The transfer characteristic for a ratio of 20% is shown in Fig. 8l. Other ratio values were tested but yielded no significant differences. At this composition, the electrolytic resistance is only slightly greater than the electrical resistance of the channel. In this setup, a few factors work against the observation of an OECT effect. First, the electrolyte is at very low concentration (10 mM). Second, the gate is uncoated and of identical diameter compared to the gold wires: the coated wires and CPD have a higher surface area, and the limited area of the gate therefore prevents additional ions from entering the bulk of the CPD. Despite these limitations, a transistor-like modulation is observed in both apparent accumulation (*V*_G_ < 0 V) and depletion (*V*_G_ > 0 V) modes. Visible signs of faradaic activity in the gate current only appear outside the [−100 mV, +300 mV] potential window and remain lower than the drain current modulation, indicating a transistor-like effect of EDOT-S-based CPDs, achieved without the use of a supporting electrolyte in the aqueous growth medium.

## 4 Conclusions

CPD morphogenesis in water was studied by quantitatively varying the major components required for the growth of electrogenerated dendrites (Table 2). CPD growth requires the presence of ions from tenths of micromolars to tenths of millimolars, with their chemical nature greatly impacting growth kinetics and morphology. Even in only a minor amount compared to the monomer, a redox agent is always required to control growth: introducing this agent can trigger the growth mechanism. The addition of a cosolvent may also affect growth in water. Monomers must be present at sufficiently high concentrations to enable growth: at least 3 mM under the specified experimental conditions. Since the solubility of EDOT is limited in water, hydrophilic EDOT derivatives may be involved in the growth process. While the effect of a glycol chain on EDOT does not promote significant changes in growth morphology and kinetics, a sulfonated chain promotes self-doping of the CPDs, enabling a genuine transistor effect without involving ions other than the monomer salt itself. This study shows that CPD morphogenesis in water is highly versatile and adaptable to diverse aqueous environments, mimicking a natural adaptive mechanism for growing transistors in a drop of water, with minimal resources and outside a cleanroom.

**Table 2:** Synoptic table summarizing the different effects observed in CPD growth in water

Features to achieve	Chemical conditions to control	Applicative aim
Growing CPDs in buffered media	Ion concentration limits growth kinetics and polymers conductance. Using an anionic electroactive monomer can compensate low medium salinity	Interfacing electronics with living systems in physiological fluids. Implementing evolutive functionalities *in situ* within electrochemical systems
Growing CPDs through a physical matrix	Growth in highly viscous liquids is kinetically limited. Possible materials of interest may include colloidal systems such as gels, as long as the continuous phase remains permeable to particle migration	Electrically interconnecting *in vitro* cell culture in a jellified Petri dish. Developing lithographically structurable electrolyte media for 3D electronic chip microfabrication
Downsizing CPDs	The nature of an electrolyte has an important influence on the morphology, ranging from dendrites to filaments. Alternatively, using different monomer structures also leads to high morphological control	Single cell electrical contacting for electrophysiology without a patch clamp. High density 3D interconnectivity in electronics and implementing filamentary switching
Modulating the temporality for CPD growth	All parameters: BQ concentration, solvent viscosity, and monomer and electrolyte concentration and nature have a direct impact on growth kinetics. The nature of the anion seems to have the most significant impact	Interconnecting living organisms within the time window of viability. Time required to train an evolving neuromorphic system
Chemically triggering CPD growth	Introducing BQ, even in trace amounts, can trigger growth in an electroactive medium, as long as enough monomers are introduced	Molecular-gating of artificial electrical synapses. Chemical transduction of evolving biosensors
Growing OMIECs in natively ion-free environments	Using an anionic monomer to grow CPDs in low electrolyte concentration	Growing OECTs off the cleanroom back-end or in a biological lab

## Conflicts of interest

The authors declare no competing interests.

## Data Availability

The data that support the findings of this study are available within the article.
